# Uterine angioleiomyoma with disseminated intravascular coagulation: a case report

**DOI:** 10.1186/s12905-023-02292-5

**Published:** 2023-04-03

**Authors:** Hanako Sato, Kosuke Murakami, Risa Fujishima, Tomoyuki Otani, Kazuko Sakai, Kazuto Nishio, Noriomi Matsumura

**Affiliations:** 1 Department of Obstetrics and Gynecology, Kindai University Faculty of Medicine, 377-2, Ohnohigashi, Osakasayama, Osaka Japan; 2 Department of Pathology, Kindai University Faculty of Medicine, Osakasayama, Osaka Japan; 3 Department of Pathology, Yachiyo Hospital, Anjo, Aichi Japan; 4 Department of Genome Biology, Kindai University Faculty of Medicine, Osakasayama, Osaka Japan

**Keywords:** Uterine angioleiomyoma, Disseminated intravascular coagulation, *CCND2*, Androgen receptor, Vascular leiomyoma

## Abstract

**Background:**

Uterine angioleiomyoma is benign tumor that composed of smooth muscle cells and thick-walled vessels. It is a very rare condition reported to present as lower abdominal mass, accompanied by dysmenorrhea and hypermenorrhea. However, its clinical presentation is not known.

**Case presentation:**

We report the case of a 44-year-old Japanese woman who developed severe anemia with disseminated intravascular coagulation without obvious external bleeding. The patient had a huge abdominal mass of over 20 cm in size, which was thought to be a uterine tumor. She received daily blood transfusions and her condition improved rapidly after she underwent hysterectomy. Pathological examination of the tumor revealed spindle-shaped cells with little atypia and mitosis, and numerous large vessels with smooth muscle and thrombus in the vessels.

**Conclusions:**

Uterine angioleiomyoma was identified as the cause of the coagulation abnormality. *CCND2* and *AR* gene amplification was detected in the tumor. Uterine tumors that present with coagulopathy despite a clinical course suggestive of benign disease should undergo differential diagnosis for uterine angioleiomyoma.

**Supplementary Information:**

The online version contains supplementary material available at 10.1186/s12905-023-02292-5.

## Background

Uterine angioleiomyoma is a benign tumor composed of smooth muscle cells and thick-walled vessels [1]. There are no reports on the incidence of this disease, but it is expected to be very rare. Uterine angioleiomyoma presents as a lower abdominal mass accompanied by dysmenorrhea and hypermenorrhea. However, there are very few case reports on uterine angioleiomyoma, and its clinical features and genetic mutations are unknown.

Here, we report a case of a patient with a uterine angioleiomyoma who developed disseminated intravascular coagulation (DIC) and rapidly improved after hysterectomy. In addition, gene panel test of the tumor was performed.

## Case presentation

A 44-year-old Japanese woman, gravida 6, para 2, was treated for mild anemia at a medical clinic. The patient complained of a slowly growing abdominal mass and had a history of surgery for ectopic pregnancy. She had no specific family history. The patient had been diagnosed with uterine fibroids 10 years earlier but had not been followed up. Her menstruation was regular, with a cycle of 27–28 days, and she did not have dysmenorrhea or significant hypermenorrhea.

The patient was referred to our hospital after routine blood tests at the medical clinic revealed severe microcytic hypochromic anemia with hemoglobin (Hb) level of 6.9 g/dL, a mean corpuscular volume (MCV) of 76 fL, and hematocrit level of 20.9%. She reported no genital bleeding. Repeated blood tests revealed a fibrinogen (Fib) level of 122 mg/dL, a fibrin/fibrinogen degradation products (FDP) level of 428.6 µg/mL, a thrombin-antithrombin complex level of 58.9 ng/mL, a plasmin-alpha2-plasmininhibitor-complex level of > 14 µg/mL, and markedly abnormal coagulation. Platelet counts (PLT), prothrombin time-international normalized ratio (PT-INR), and activated partial thromboplastin time (APTT) were within normal range. The patient was suspected of having coagulation abnormalities caused by leukemia but a subsequent bone marrow examination revealed no abnormal findings.

**Fig. 1:**
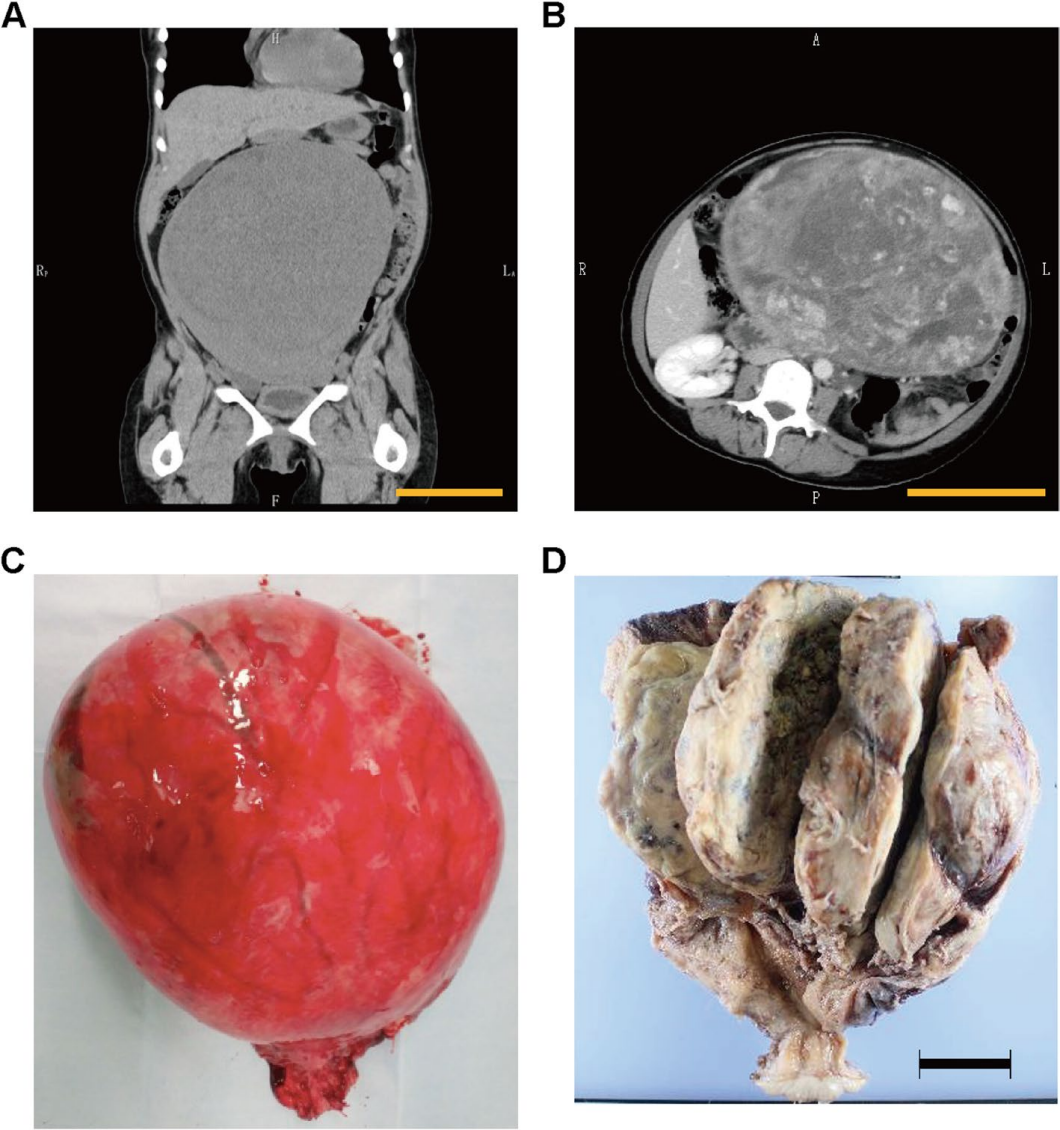
Imaging and resected specimen. **A**. Plane computed tomography showed a giant pelvic mass (23 cm). scale bar: 10 cm. **B**. Contrast-enhanced CT showed heterogeneous contrast inside the tumor. scale bar: 10 cm. **C** & **D**. The mass was not in continuity with the endometrium and originated within the myometrium. scale bar: 5 cm

Seven days after the patient’s first visit to our hospital, her condition continued to deteriorate (Fib: 99 mg/dL) and a uterine tumor was suspected. A gynecological examination 14 days after her initial visit revealed that her uterus was soft and swollen to the size of an adult head. Ultrasonography revealed a highly echogenic, substantial mass in the pelvis. A computed tomography (CT) scan revealed a large pelvic mass (23 × 16 cm, adjacent to the uterus) with contrast enhancement and a heterogeneous interior (Fig. 1A–B). Magnetic resonance imaging (MRI) scan could not be performed because of the patient’s panic disorder and claustrophobia. Blood tests revealed anemia with hemolysis and thrombocytopenia, with a Hb level of 8.3 g/dL, MCV level of 87.2 fL, a reticulocyte count of 3.7%, and a PLT count of 10.8 × 10^4^ /µL. Blood tests did not show any crushed erythrocytes, and the indirect Coombs test results were negative. Analysis of the coagulation system revealed a Fib level of 94 mg/dL, an FDP levels of 374.5 µg/mL, a thrombin-antithrombin complex (TAT) level of 59.4 ng/mL, a plasmin-alpha2-plasmininhibitor-complex (PIC) level of > 14 µg/mL, and a d-dimer level of 81 µg/m. The levels of PT-INR, APTT, antithrombin-III, and lactate dehydrogenase (203 U/L) were within normal ranges. Tumor markers, CA19-9 and CA125, were also within normal ranges. Contrast-enhanced CT showed no evidence of deep venous thrombosis or pulmonary embolism. The patient scored 4 DIC points based on the international society of thrombosis and hemostasis criteria [2], and 7 DIC points based on Japanese society of thrombosis and hemostasis criteria [3]. The patient was therefore diagnosed with DIC and transfused with 4U of fresh frozen plasma (FFP) and 10U of PLT. She was urgently admitted to the hospital the following day for treatment.

**Fig. 2:**
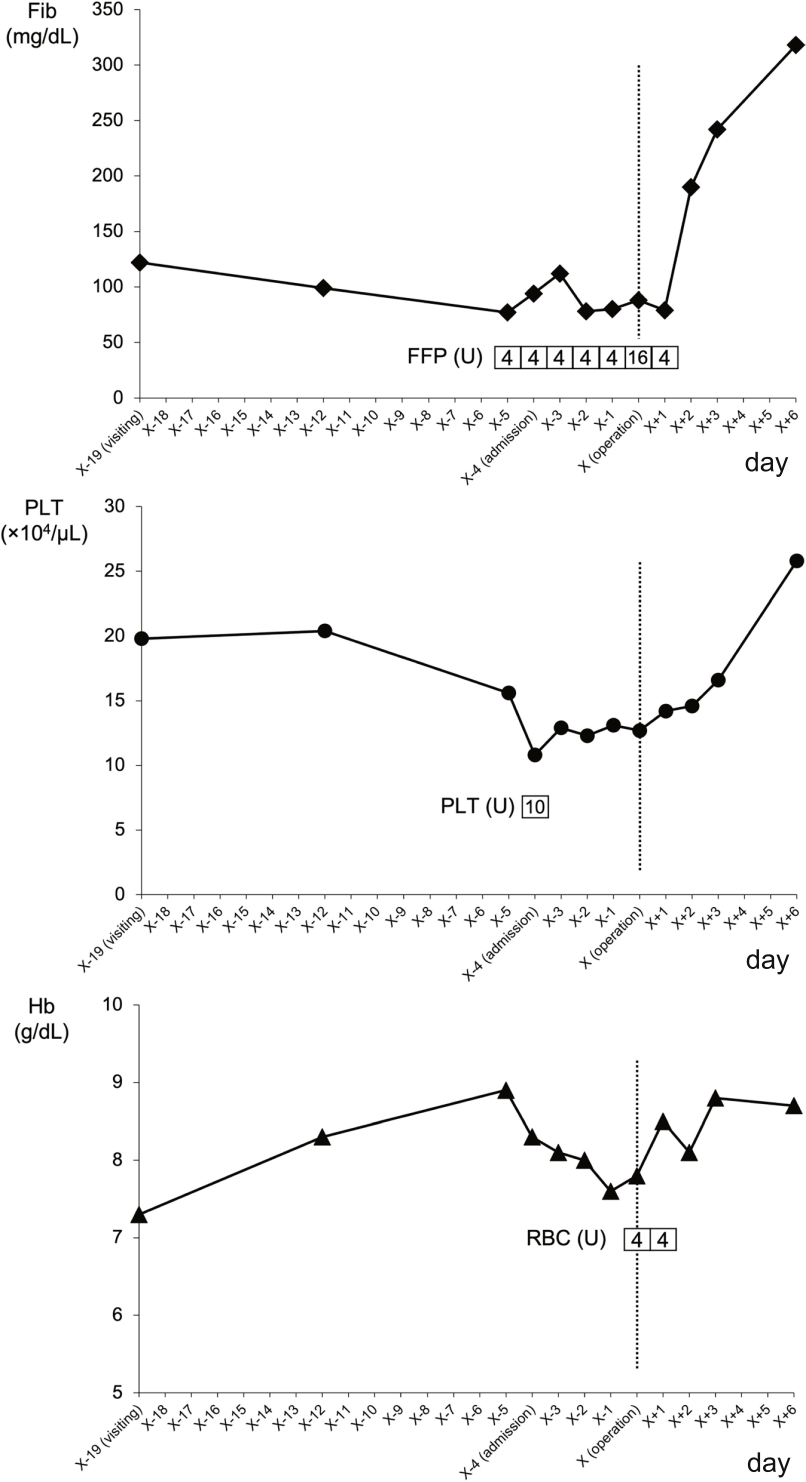
Change of fibrinogen (Fib), platelet (PLT), and hemoglobin (Hb) levels. X axis; Day. The date of operation is X. Y axis; data level. The vertical dotted lines indicate the date of operation

Despite receiving daily transfusions of FFP, the patient’s coagulation factors improved poorly. On the sixth day after admission, she underwent abdominal total hysterectomy and bilateral oophorectomy under general anesthesia, which revealed a swollen uterus the size of an adult head. No adhesions, ascites effusions, dissemination, or other signs of malignancy were observed in the abdominal cavity. The intraoperative blood loss was 1195 ml. The tumor weighted 2900 g. The tumor, which was not continuous with the endometrium, originated from within the myometrium (Fig. 1C–D). Coagulation factor abnormalities improved soon after the surgery and blood transfusions were no longer required. The patient progressed well and was discharged on postoperative day 8. On postoperative day 12, she was treated with antibiotics for a vaginal cutaneous abscess, which improved. Figure 2 shows the trends in Fib, PLT, and Hb levels, as well as blood transfusions.

**Fig. 3:**
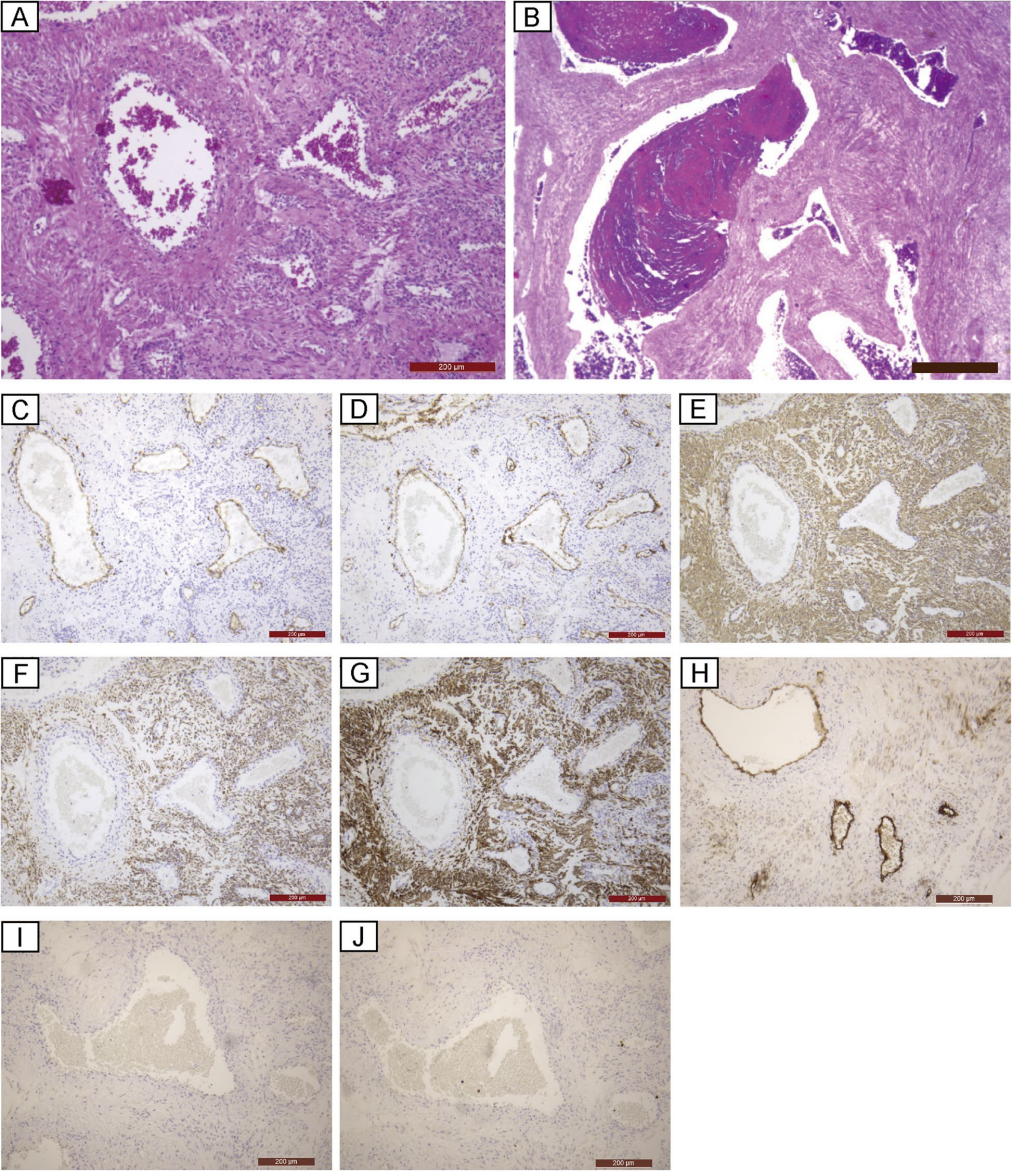
Pathological findings. **A**. Hematoxylin–Eosin staining. There are spindle cells with eosinophilic sporangia and numerous large blood vessels with smooth muscle. Magnification: 100 × , scale bar: 200 µm. **B**. Hematoxylin–Eosin staining. Organized thrombi are seen. Magnification: 25 × , scale bar: 800 µm. **C**. CD31. Magnification: 100 × , scale bar: 200 µm. **D**. CD34. Magnification: 100 × , scale bar: 200 µm. **E**. α-SMA. Magnification: 100 × , scale bar: 200 µm. **F**. Desmin. Magnification: 100 × , scale bar: 200 µm. **G**. Caldesmon. Magnification: 100 × , scale bar: 200 µm. **H**. CD10. Magnification: 100 × , scale bar: 200 µm. **I**. HMB-45. Magnification: 100 × , scale bar: 200 µm. **J**. MIB-1. Magnification: 100 × , scale bar: 200 µm

Histopathologic analysis (hematoxylin and eosin) revealed a flowing array of spindle-shaped cells with eosinophilic sporangia, without atypia or schistosomiasis. The tumor had numerous large vessels with smooth muscle and thrombus within the vessels (Fig. 3A–B). The tumor also showed partial microscopic necrosis, but no intravascular leiomyoma was observed. Immunohistochemical analysis showed that the thick vascular endothelium was positive for CD31 and CD34, and that the spindle-shaped cells were positive for α-SMA, Desmin, and Caldesmon (Fig. 3C–G). The tumor was partially positive for CD10, negative for HMB-45, and had approximately 2% MIB-1 positivity (Fig. 3H–J). Based on these findings, the tumor was diagnosed as uterine angioleiomyoma. The gene panel test revealed the amplification of *CCND2* (copy number: 5) and *AR* (androgen receptor; copy number: 5.14) (Supplementary Table 1 and Supplementary Fig. 1).

## Discussion and conclusions

To our knowledge, 41 cases of uterine angioleiomyoma have been reported so far [4] [5]. The patient was found to have coagulation factor-consuming DIC triggered by anemia without genital bleeding, which was thought to be due to uterine angioleiomyoma. This is the first reported case of uterine angioleiomyoma discovered in this context to be reported worldwide.

Uterine angioleiomyoma presents as an abdominal mass [6], some large cases exceeding 30 cm have been reported [7]. In this case, the tumor was also quite large, and the patient was aware of the sensation of an abdominal mass. Uterine angioleiomyoma may also cause excessive menstruation and abnormal genital bleeding due to uterine enlargement and numerous vascular growths [6]. There have been case reports of spontaneous rupture of uterine angioleiomyoma causing massive hemorrhage [8]. However, in the present case, the patient did not have excessive menstruation, dysmenorrhea, or difficulty in daily activities.

There have been case reports of uterine angioleiomyoma causing excessive vaginal bleeding and consumptive coagulopathy [9], but in these case reports, it is not clear whether the massive genital bleeding was caused by the consumptive coagulopathy or whether the genital bleeding caused the consumptive coagulopathy. On the other hand, there was no event of massive genital bleeding in this case. And the hemoglobin level, which had not recovered after massive blood transfusions despite the absence of external bleeding preoperatively, recovered quickly after hysterectomy and no longer required blood transfusions. The coagulation system also improved promptly after surgery. Pathological findings also showed numerous large blood vessels within the tumor and thrombus within the vessels. A mechanism has been proposed in which stagnation of blood in tumor vessels leads to thrombus formation, and ischemic damage releases procoagulant factors that consume red blood cells, coagulation factors, platelets, and thrombin [10, 11]. These clinical and histopathological findings strongly suggest that the consumption of coagulation factors in the tumor vessels may have led to DIC. Surgical removal of the tumor is considered important in the treatment of this condition.

Although MRI can effectively detect uterine angioleiomyoma, it could not be performed in this case. Although it is extremely difficult to diagnose uterine angioleiomyoma based on CT scans, contrast-enhanced CT can reveal the following: (1) the presence of multiple vascular branches within the tumor, (2) late-phase, heterogeneous "sand-like" enhancement, and (3) large bilateral pelvic varicocele and uterine arterial hypertrophy [7]. In this case, contrast-enhanced CT shows "sand-like" enhancement (Fig. 1B), suggesting uterine angioleiomyoma.

To our knowledge, this is the first case of genetic testing was done for uterine angioleiomyoma. *MED12* mutations are present in approximately 70% of uterine leiomyomas, which also carry mutations in genes involved in smooth muscle formation, such as *COL4A6*, *DCN*, and *AHR*, as well as genetic abnormalities in *HMGA2* and *FH* [12]. In this case, *MED12* mutation, which is the most common in uterine leiomyomas, was not detected. On the other hand, the gene panel test performed in this case did not include *COL4A6*, *DCN*, *AHR*, *HMGA2*, or *FH*. More detailed genetic testing may be needed to determine the difference in genetic background between angioleiomyoma and leiomyoma.

In conclusion, slow-growing uterine tumors should be considered in the differential diagnosis of uterine angioleiomyoma if they present with coagulopathy despite a clinical course suggestive of benign disease. The background of genetic mutations in uterine angioleiomyoma might be different from that in uterine leiomyoma, however, further studies are needed.

## Supplementary Information

**Additional file 1:** **Supplemetary Table 1.** Gene panel test.

**Additional file 2: Supplementary Fig.1.** Plot of copy number per amplicon. The copy number per amplicon is plotted by log2 ratio. CCND2 (chromosome 11) and AR (chromosome X) are indicated by red dots. The region length of each chromosome does not reflect the actual chromosome length due to targeted amplicon sequencing.

## Data Availability

The datasets used and/or analysed during the current study available from the corresponding author on reasonable request.

## Abbreviations

DIC: Disseminated intravascular coagulation
Hb: Hemoglobin
MCV: Mean corpuscular volume
Fib: Fibrinogen
FDP: Fibrin/fibrinogen degradation products
PLT: Platelet counts
PT-INR: Prothrombin time-international normalized ratio
APTT: Activated partial thromboplastin time
CT: Computed tomography
MRI: Magnetic resonance imaging
TAT: Thrombin-antithrombin complex
PIC: Plasmin-alpha2-plasmininhibitor-complex
FFP: Fresh frozen plasma
